# Predicting HIV self-testing intentions among Chinese college students: a dual-model analysis integrating health belief constructs and machine learning prioritization

**DOI:** 10.3389/fpubh.2025.1596876

**Published:** 2025-07-31

**Authors:** Yan Jiang, Jing Li, Jingfen Lu, Liping He, Lin Hu, Jiazhen He, Xianli Huang, Yuchao Li

**Affiliations:** ^1^School of Public Health, Xiangnan University, Chenzhou, China; ^2^Department of Public Health and Preventive Medicine, School of Medicine, Jinan University, Guangzhou, China; ^3^The First Clinical Medical College, Guangzhou University of Chinese Medicine, Guangzhou, China; ^4^College of Education Hunan University of Humanities, Science and Technology, Loudi, China

**Keywords:** health belief model, college student, HIV self-testing, high-risk behaviors, random forest modeling

## Abstract

**Introduction:**

As college students emerge as a key HIV-vulnerable population in China, HIV self-testing (HIVST) presents a critical strategy for enhancing detection rates and enabling timely intervention. While observational studies have identified multifactorial influences on HIVST willingness, few investigations integrate behavioral theory with machine learning approaches among college students. This study aims to fill this gap by exploring the determinants of HIVST willingness among college students using the Health Belief Model (HBM) and random forest analytics.

**Methods:**

This cross-sectional study employed stratified cluster sampling to recruit 1,015 undergraduates from Xiangnan College (July-August 2022), The Health Belief Model (HBM) was synthesized with random forest analytics to elucidate determinants of HIVST willingness. Data were collected through questionnaires, and logistic regression and random forest modeling were used for analysis.

**Results:**

Among participants, 69.3% (*n* = 703) expressed willingness to adopt HIVST within the next 6 months. 15.0% reported sexual activity (*n* = 152), with 12.0% (*n* = 122) of sexually active participants demonstrating concurrent engagement in unprotected intercourse and HIV testing willingness. HBM-based logistic regression revealed that self-efficacy (OR = 1.64, 95% CI: 1.21-2.21) and cues to action (OR = 1.34, 1.04-1.75) were significant facilitators, contrasting with the inhibitory effects of perceived barriers (OR = 0.69, 0.55-0.86). Random forest modeling prioritized these psychological constructs (mean decrease Gini >2.5), identifying male students and arts majors as critical subpopulations requiring targeted intervention.

**Discussion:**

Our dual-method analysis establishes that campus HIV control necessitates: 1) Gender-specific prevention programs addressing male students’ elevated risk exposure; 2) HBM-informed education strengthening self-efficacy and environmental cues; 3) Structural interventions reducing testing barriers through discreet service delivery. This theoretical-empirical integration advances predictive understanding of HIVST behaviors, providing actionable insights for developing precision public health strategies in academic settings.

## Introduction

Acquired Immunodeficiency Syndrome (AIDS), caused by the human immunodeficiency virus (HIV), has been a major global public health challenge, and its prevalence has had a significant impact on the sexually active youth population, especially college students (1). According to the Joint United Nations Programme on HIV/AIDS (UNAIDS), there was an average of 3,200 new HIV infections each day among adults aged 15 and older, with approximately 30% of these infections occurring in individuals aged 15–24 in 2022 (2). Worryingly, the number of new HIV infections among college students in China has been growing sharply in recent years, increasing at an annual rate of 30–50% (3). Furthermore, the proportion of students among reported HIV cases has risen from 8.5% in 2010 to 21.7% in 2019, with 98.2% of these cases resulting from sexual transmission (4). This alarming trend indicates that college students have become a high-risk group for HIV infection. However, HIV testing rates among sexually active Chinese university students remain significantly low. For example, a study in Chongqing found that only 17.16% of adolescents aged 14–25 years with a history of sexual activity had undergone HIV testing (5). Moreover, a nationwide survey revealed substantial knowledge gaps among university students regarding HIV treatment, self-testing, and post-exposure prophylaxis (6).

During emerging adulthood, Chinese university students exhibit distinct developmental and social characteristics that profoundly influence their HIV risk and testing behaviors (7–10). Arnett’s theory identifies this stage as one of identity exploration, instability, and self-focus, with students often feeling “caught in the middle” between adolescence and full adulthood (11). In China, these characteristics are further complicated by cultural norms such as family honor and sexual conservatism. For example, while students may explore their sexual orientation and relationships in the relatively free environment of universities, they often face internal conflict due to the evolving societal acceptance of non-heterosexual identities (12). High academic pressure, future uncertainties, and strong family ties also shape their health decisions (13, 14). Moreover, the dense social networks and fluid sexual behaviors in universities increase the risk of HIV transmission (15–17). Collectively, these factors create a complex decision-making psychology around HIV testing.

Although HIV self-testing (HIVST) has emerged as an important preventive measure and early detection tool (18, 19), a key barrier contributing to this testing insufficiency remains the persistent stigma surrounding HIV/AIDS continues to prevent at-risk populations from utilizing traditional testing (20, 21) services. This stigma is particularly pronounced among adolescents, who are among the most vulnerable to HIV infection due to their earlier initiation of sexual activity and subsequent unsafe sexual behaviors, such as having multiple sex partners, engaging in casual or commercial sex, and inconsistent condom use (10, 22–24). For example, a cross-sectional study based on a large national sample of 18,000 Chinese college students (10) revealed that nearly one-third of sexually active college students initiated sexual activity before the age of 18. Meanwhile, many individuals are less inclined to undergo testing because they perceive themselves as being at low risk of contracting a Sexually Transmitted Infection (STI) (25, 26). These behaviors, combined with the social stigma surrounding HIV/AIDS, further exacerbate the challenges faced by adolescents in accessing HIV testing and prevention services. Moreover, traditional attitudes toward sexuality in Chinese society significantly impact the acceptance of HIV testing among this age group (27). For instance, the prevalence of HIV among men who have sex with men (MSM) in China is significantly higher than in the general population (28). However, HIV testing acceptance among this group remains low, partly due to the negative social attitudes and discrimination that MSM face (29). These cultural and social barriers create significant challenges for HIV prevention and testing efforts, limiting the effectiveness of traditional testing services. In this context, HIVST offers a promising solution by bypassing the barriers associated with facility-based testing, such as social stigma and geographic accessibility constraints (30–32). This approach not only addresses the unique challenges faced by adolescents and high-risk groups but also enhances overall HIV prevention strategies in the Chinese cultural context.

From a social psychological perspective, the Health Belief Model (HBM) offers a robust framework for analyzing health decision-making through six core constructs: perceived susceptibility, severity, benefits, barriers, self-efficacy, and cues to action (33). These constructs collectively inform the health decision-making process. Although the HBM has been extensively applied in HIV-related behavioral research (34–36), current studies on HIVST adoption exhibit two key limitations: first, a predominant focus on the general population rather than specific behavioral subgroups (37, 38); and second, insufficient exploration of high-risk college student populations with documented unprotected sexual exposure (39, 40). This gap impedes the development of culturally competent, targeted interventions for those most vulnerable to HIV infection.

To more accurately identify the key factors influencing HIV testing and diagnosis among Chinese university students and to predict related behaviors, this study employed a random forest algorithm to rank the importance of these factors. Unlike conventional methods such as logistic regression (41), machine learning algorithms have demonstrated significant strengths in handling high-dimensional, complex, and potentially non-linear health behavior data. They are particularly well-suited for exploring behavioral prediction in the context of multifactorial interactions (42). In the field of public health, especially in HIV/AIDS research, these models have been successfully applied to predict infection risk, identify at-risk populations, optimize the targeting of interventions, and understand health service utilization patterns (42, 43). For example, He et al. utilized sentinel surveillance data of MSM individuals in Zhejiang province to predict HIV infection risk, and the random forest algorithm achieved the best performance (AUC = 0.846) (43). However, there are relatively few applications for high-risk Chinese college students.

To address these gaps, our study has three main objectives: (1) applying HBM constructs to investigate HIVST willingness among college students; (2) employing random forest modeling to identify key behavioral determinants; (3) establishing an evidence base for precision interventions targeting high-risk collegiate subgroups. This dual theoretical-empirical approach aims to enhance early HIV detection rates and inform tertiary prevention strategies tailored to the behavioral realities of university populations.

## Methods

### Study design and population

This cross-sectional investigation was conducted among undergraduate students at Xiangnan University during July–August 2022. In this study, we employed a stratified whole-cluster random sampling method. Universities were divided into four strata based on discipline type: science and engineering, humanities, arts and sports, and medicine. Using computer-generated random numbers, we selected two representative majors from each stratum to ensure representativeness. For each selected major, we compiled a list of all eligible classes and randomly chose two complete classes as study clusters. This ensured that two classes from each major participated in the study. The sample size was determined based on study objectives, feasibility, and uniform class sizes (typically 30–50 students) to ensure comparability across disciplines. While not all majors had the same number of classes, and some had more than the predetermined number, we ensured all eligible classes were included in the sampling frame and had an equal chance of selection. This study adhered to the STROBE (Strengthening the Reporting of Observational Studies in Epidemiology) guidelines for reporting observational research.

### Research procedures

Participants were invited to complete an online survey via a link forwarded by their class president through Wenjuanxing.^1^ This method was chosen due to its suitability for a large population, as well as the time and cost-saving advantages it offers. The survey was designed to be concise, taking approximately 2 min to complete, to minimize the burden on participants. Confidentiality and anonymity were assured, with the platform automatically recording response times and ensuring data security. Participants provided informed consent by selecting the “I agree” button after reviewing the terms and conditions of the study; those who selected “I do not agree” were automatically exited from the survey without any information being stored. Participants could also withdraw from the study at any point by selecting a “quit” button on each page of the survey.

To thank participants for their cooperation, a small gift (e.g., a notebook or stationery set) was provided upon completion, distributed by the class president. The research team regularly reviewed the returned questionnaires to exclude any that did not meet the study’s requirements. The study was approved by the ethics committee of Xiangnan College, and all participants obtained informed consent.

### Sample size determination

The minimum required sample size was calculated using the standard formula for cross-sectional studies: *n=* $Z_{1-\alpha /2}^{2}\times \frac{p(1-p)}{d^{2}}$ . Where *Z* = 2.576 (corresponding to 99% confidence level), *p* = 0.624 [expected self-test rate based on prior research (44)], and *d* = 0.0624 (10% of *p* as permissible error). This yielded an initial estimate of 397 participants. Accounting for potential sampling error and non-response bias, we adjusted the target sample size to 874 participants. The adjustment process was as follows: we anticipated an invalid response rate of approximately 10%, necessitating an increase in the sample size by 10%. Additionally, considering the design effect of stratified sampling, the final sample size was calculated as 397 × (1 + 0.1) × 2 = 873.4, which we rounded up to 874 participants. However, during the data collection process, we were able to exceed this target and successfully gather data from 1,032 individuals.

### Participant selection criteria

A total of 1,032 initial samples were collected for this study. The eligibility criteria for participation were: (a) current university students, and (b) aged 18 or above. The exclusion criteria were: (a) overseas exchange students, and (b) individuals with severe cognitive impairment. All 1,032 samples met the eligibility criteria and did not meet the exclusion criteria.

### Research variables and measurement tools

#### Demographics

Demographic characteristics included age, gender, specialized field, grade, household registration, and living expenses.

Behavioral Characteristics and HIV-Related Knowledge.

It included relationship status, sexual orientation, number of sexual partners, and knowledge of HIV.

#### The Health Belief Model

The research instrument was developed through a rigorous process guided by the HBM framework. Following initial item generation based on HBM constructs, we conducted a pilot study with 50 participants to refine question clarity and content validity. This iterative process resulted in a finalized questionnaire comp rising six validated domains (27 items total): Perceived Susceptibility (3 items; dichotomous scale); Perceived Severity (7 items; dichotomous scale); Perceived Benefits (4 items; 5-point Likert); Perceived Barriers (5 items; 5-point Likert); Self-Efficacy (4 items; 5-point Likert); Cues to Action (4 items; 5-point Likert). These six domains correspond to the core constructs of the HBM. HBM threat appraisal components (susceptibility/severity) employed binary scoring (1 = No, 2 = Yes). Positive construct scales (benefits, self-efficacy, cues to action) used conventional Likert anchoring (1 = Strongly Disagree to 5 = Strongly Agree). Barrier items applied reverse scoring to maintain directional consistency. The instrument demonstrated satisfactory reliability with an overall *Cronbach’s α* coefficient of 0.837. Content Validity was formally established through independent evaluation by 5 Experts in HIV Prevention and Control. All items demonstrated excellent relevance (I-CVI = 0.80–1.00), with a perfect Scale-CVI (S-CVI = 1.0) indicating unanimous expert consensus on item essentiality. Construct Validity was assessed via exploratory factor analysis (EFA). The Kaiser-Meyer-Olkin measure (KMO = 0.926) confirmed sampling adequacy, and Bartlett’s test of sphericity (χ^2^ = 28296.716, *p* < 0.001) supported the factorability of the data. A single-factor solution accounted for 72.834% of the total variance, validating the hypothesized unidimensional structure. Specific entries and detailed information are presented in Supplementary Method 1 and Supplementary Table S1.

#### Definition of willingness to self-test

The outcome variable was defined as willingness to self-test for HIV. This was assessed using two yes/no questions: (1) “Have you ever had unprotected sex with a sexual partner?” and (2) “Willingness to undergo HIVST in the next 6 months?” Participants who answered “yes” to both questions were classified as being willing to self-test for HIV.

### Machine learning

To evaluate the importance of each feature, we employed a random forest classifier comprising 500 trees with mtry = √*p*. We quantified variable importance using the Mean Decrease in Gini (MDG), measuring the average reduction in Gini impurity attributed to each feature across all decision trees. A higher MDG value indicates that the feature effectively increases node purity when partitioning the data, thereby enhancing the model’s predictive accuracy. We validated model performance through 10-fold cross-validation, complementing this with receiver operating characteristic (ROC) analysis to assess predictive accuracy. We defined statistical significance as *p* < 0.05 (two-tailed), and we conducted all analyses using the Random Forest package.

### Statistical analysis

Using the web-based survey platform Wenjuanxing (see text footnote 1), we performed data management and statistical analyses with embedded quality controls, including real-time completeness checks and range validations. Using R v4.3.2 (R Foundation for Statistical Computing), we performed all statistical procedures. We characterized continuous variables by mean ± SD (for normally distributed variables, as determined by the *Shapiro–Wilk test*) or median [IQR], and expressed categorical variables as frequencies (%). Intergroup comparisons utilized Pearson’s *χ*^2^*/Fisher’s* exact tests for categorical variables and independent *t-tests*/*Wilcoxon rank-sum tests* for continuous variables. Multivariable logistic regression with backward stepwise selection (retention *p* < 0.10) identified determinants of HIVST willingness, reporting adjusted odds ratios (*ORs*) with 95% confidence intervals (*CIs*), validated through Hosmer-Lemeshow goodness-of-fit (*p* > 0.05) and variance inflation factors (*VIF* <5) (Supplementary Table S2).

## Results

### Participants characteristics

During the data collection process, 10 respondents voluntarily withdrew, and 7 submitted incomplete questionnaires (with a completion rate of less than 50%), resulting in a final sample of 1,015 valid responses out of 1,032 collected questionnaires, yielding a validity rate of 98.4%. Supplementary Figure S1 illustrates the participant enrollment flowchart with detailed inclusion/exclusion processes. Participants comprised undergraduates aged 17–24 years (mean age 19.9 ± 1.3 years), predominantly female (66.6%, *n* = 676), with freshmen/sophomores constituting 69.5% of the cohort. Over half majored in humanities/history or medical disciplines (53.2%), 54.0% held urban household registrations, and 55.0% reported monthly disposable income levels of ¥1,000-1,500. Most participants were single (73.8%), with limited sexual partner diversity (≥3 partners: 6.5%). Regarding HIV-related characteristics, 57.2% had received HIV/AIDS prevention education, 15.0% (*n* = 152) reported unprotected sexual intercourse, and 45.6% (*n* = 463) had undergone prior HIV testing (Table 1).

**Table 1:** Comparison of HIV self-testing rates between different socio-demographic characteristics (n, %).

Characteristic	Total	Ever had unprotected sex	Have unprotected sex and be willing to take an HIV test	Willingness to HIV self-testing in the next 6 months	*χ* ^2^	*P* -value
Participants	1,015 (100.0)	152 (15.0)	122 (12.0)	703 (69.3)		
Gender					5.524	0.019
Male	339 (33.4)	71 (20.9)	58 (17.1)	218 (64.3)		
Female	676 (66.6)	81 (20.0)	64 (9.5)	485 (71.7)		
Specialized field					6.680	0.083
Science and engineering	204 (20.1)	19 (9.3)	16 (7.8)	138 (67.6)		
Literature and history	375 (36.9)	46 (12.3)	38 (10.1)	251 (66.9)		
Art	100 (9.9)	35 (35.0)	24 (24.0)	64 (64.0)		
Medical	336 (33.1)	52 (15.5)	44 (13.1)	250 (74.4)		
Grade					3.999	0.262
Freshman	277 (27.3)	49 (17.7)	36 (13.0)	200 (72.2)		
Sophomore	428 (42.2)	70 (16.4)	61 (14.3)	301 (70.3)		
Junior	225 (22.2)	19 (8.4)	14 (6.2)	145 (64.4)		
Senior/Fifth	85 (8.4)	14 (16.5)	11 (12.9)	57 (67.1)		
Household registration					0.173	0.677
Countryside	467 (46.0)	67 (14.3)	52 (11.1)	327 (70.0)		
Cities and towns	548 (54.0)	85 (15.5)	70 (12.8)	376 (68.6)		
Living expenses, (yuan/month)					5.999	0.112
<1,000	92 (9.1)	8 (8.7)	8 (8.7)	65 (70.7)		
1,000~	558 (55.0)	60 (10.8)	45 (8.1)	370 (66.3)		
1,500~	275 (27.1)	50 (18.2)	44 (16.0)	205 (74.5)		
≥2000	90 (8.9)	34 (37.8)	25 (27.8)	63 (70.0)		
Received relevant knowledge on AIDS prevention and treatment					1.500	0.221
Yes	581 (57.2)	55 (9.5)	46 (7.9)	393 (67.6)		
No	434 (42.8)	97 (22.4)	76 (17.5)	310 (71.4)		
Relationship status					15.035	0.001
Single	749 (73.8)	71 (9.5)	54 (7.2)	494 (66.0)		
Have a male (female) friend	256 (25.2)	77 (30.1)	66 (25.8)	202 (78.9)		
Married	10 (1.0)	4 (40.0)	2 (20.0)	7 (70.0)		
Sexual orientation					3.505	0.320
Heterosexuality	854 (84.1)	134 (15.7)	107 (12.5)	591 (69.2)		
Homosexuality	24 (2.4)	4 (16.7)	3 (12.5)	18 (75.0)		
Bisexuality	53 (5.2)	11 (20.8)	11 (20.8)	41 (77.4)		
I do not know	84 (8.3)	3 (3.6)	1 (1.2)	53 (63.1)		
Number of sexual partners					17.945	<0.001
0	709 (69.9)	0 (0.0)	0 (0.0)	465 (65.6)		
1	173 (17.0)	87 (50.3)	67 (38.7)	134 (77.5)		
2	67 (6.6)	31 (46.3)	28 (41.8)	57 (85.1)		
≥3	66 (6.5)	34 (51.5)	27 (40.9)	47 (71.2)		
Have been tested for HIV					0.434	0.510
No	552 (54.4)	43 (7.8)	38 (6.9)	377 (68.3)		
Yes	463 (45.6)	109 (23.5)	84 (18.1)	326 (70.4)		

### Univariate analysis of HIVST willingness

Among participants, 69.3% (*n* = 703) expressed willingness to adopt HIVST within the next 6 months. 67.8% of participants (*n* = 688) reported having engaged in sexual activity, and 15.0% of participants (*n* = 152) reported having unprotected sex. Among them, 12.0% of participants (*n* = 122) reported having unprotected sex and expressed willingness to undergo HIV testing. The results of the univariate analyses showed that significantly higher HIVST willingness among female, participants with intimate partners, and individuals reporting ≥ 2 sexual partners (*p* < 0.05). Comparative analyses of sociodemographic predictors are detailed in Table 1.

### HBM and HIVST willingness

Univariate analysis of HBM dimension scores revealed significant associations with HIVST willingness (Table 2). Defined as concurrent engagement in unprotected sex and testing willingness, the outcome demonstrated differential HBM domain impacts through *ANOVA*. Perceived benefits (*t* = 2.957, *p* = 0.003), self-efficacy (*t* = 5.426, *p* < 0.001), and cues to action (*t* = 6.263, *p* < 0.001) positively predicted testing willingness, whereas perceived susceptibility, severity, and barriers showed inverse correlations (Supplementary Figure S2). Higher scores in promotive domains (benefits: *β* = 0.24; self-efficacy: *β* = 0.38; cues: *β* = 0.42) corresponded to increased testing intention, while inhibitory domains (susceptibility: *β* = −0.18; severity: *β* = −0.15; barriers: *β* = −0.31) exhibited dose-dependent suppression effects.

**Table 2:** One-way analysis of scores on HBM dimensions and willingness to HIV self-testing.

Variant	Have unprotected sex and be willing to take an HIV test		*t*-value	*p*-value
	No (*n* = 893)	Yes (*n* = 122)		
Perceived susceptibility	4.71 ± 1.31	4.67 ± 1.36	−0.419	0.675
Perceived severity	11.07 ± 2.99	11.02 ± 3.13	−0.239	0.811
Perceived benefits	16.20 ± 3.83	16.98 ± 3.90	2.957	0.003
Perceived barriers	15.92 ± 3.94	15.59 ± 4.19	−1.184	0.237
Self-efficacy	13.36 ± 2.99	14.48 ± 3.05	5.426	<0.001
Cues to action	14.40 ± 3.22	15.74 ± 3.11	6.263	<0.001

### Multivariate analysis of HIVST willingness

Multivariate logistic regression model (dependent variable: 0 = unwilling, 1 = willing) incorporated demographic, behavioral, and psychological covariates. Backward stepwise selection identified female gender (OR = 1.69, 95% CI: 1.21–2.37), intimate partnership (OR = 1.63, 1.09–2.44), and multiple sexual partners (OR = 2.75, 1.23–6.18) as positive predictors. Arts majors demonstrated reduced willingness (OR = 0.53, 0.29–0.97). HBM components revealed self-efficacy (OR = 1.64, 1.21–2.21) and cues to action (OR = 1.34, 1.04–1.75) as facilitators, while perceived barriers emerged as the strongest inhibitor (OR = 0.69, 0.55–0.86) (Table 3).

**Table 3:** Logistic regression analysis of factors influencing willingness to HIV self-testing.

Characteristic	Β	SE	Z	OR (95%CI)	*P*
Gender					
Male				1	
Female	0.525	0.173	3.040	1.69 (1.21, 2.37)	0.002
Specialized field					
Science and engineering				1	
Literature and history	−0.395	0.217	−1.826	0.67 (0.44, 1.03)	0.068
Art	−0.635	0.307	−2.069	0.53 (0.29, 0.97)	0.039
Medical	0.139	0.236	0.588	1.15 (0.72, 1.83)	0.557
Relationship status					
Single				1	
Have a male (female) friend	0.487	0.206	2.367	1.63 (1.09, 2.44)	0.018
Married	−0.039	0.802	−0.048	0.96 (0.20, 4.64)	0.962
Number of sexual partners					
0				1	
1	0.276	0.279	0.990	1.32 (0.76, 2.28)	0.322
2	1.013	0.412	2.458	2.75 (1.23, 6.18)	0.014
≥3	0.067	0.355	0.189	1.07 (0.53, 2.14)	0.850
Perceived barriers	−0.375	0.112	−3.355	0.69 (0.55, 0.86)	<0.001
Self-efficacy	0.492	0.153	3.222	1.64 (1.21, 2.21)	0.001
Cues to action	0.296	0.133	2.221	1.34 (1.04, 1.75)	0.026

### Machine learning validation

**Figure 1:**
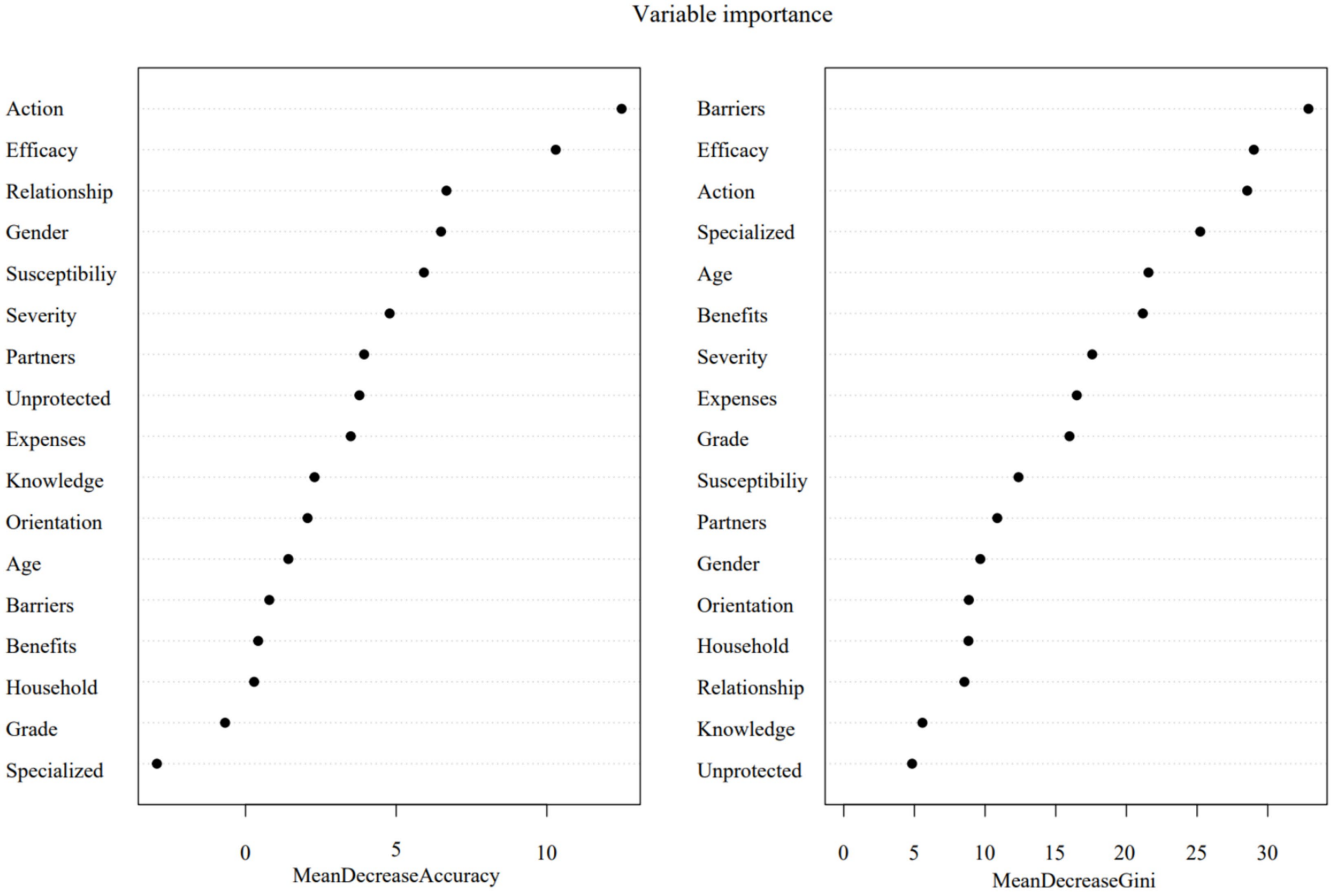
Random forest modeling of HIV self-testing intention. Action, Cues to action; Efficacy, Self-efficacy; Relationship, Relationship status; Susceptibility, Perceived susceptibility, Severity, Perceived severity; Partners, Number of sexual partners; unprotected, Ever had unprotected sex; Expenses, Living expenses; Knowledge, Received relevant knowledge on AIDS prevention and treatment; Orientation, Sexual orientation; Barriers, Perceived barriers; Benefits, Perceived benefits; Household, Household registration; Specialized, Specialized field.

Random forest analysis (500 trees, mtry = √p) prioritized variable importance through dual metrics (Figure 1): mean decrease accuracy (top predictors: cues to action, self-efficacy, relationship status, and age) and Gini impurity reduction (key determinants: perceived barriers, self-efficacy, cues to action, and specialized field). The model demonstrated excellent predictive accuracy (AUC = 0.942), with stabilized out-of-bag error rates below 0.2 beyond 150-tree iterations (Supplementary Figure S3).

## Discussion

In this study, the prevalence of unprotected intercourse was found to be 15.2%, which is significantly higher than the rates reported in Jilin (8.6%) (45) and Shandong Province (5.9%) (46), suggesting potential regional disparities in sexual health literacy. This prevalence aligns with the 10–20% range documented among university students in Western countries (e.g., United States, United Kingdom) (47, 48), indicating a global challenge in young adult populations. In addition, 45.6% of participants had undergone HIVST, a rate significantly higher than that of college students in Lianyungang City (18.4%) (49) and substantially greater than the national average of 3.1% across 222 higher education institutions (50). Additionally, 703 participants (69.3%) expressed willingness to undergo HIVST within the next 6 months, surpassing the 62.5% reported in a related study in Chengdu (51). Globally, there are significant disparities in the acceptance and utilization of HIVST. For instance, in Kenya (52), the usage rate of HIVST is as high as 52%, while in Nigeria (53), only 16% of young women and 9% of young men have ever been tested for HIV. The reason for this difference could be the installation of HIVST reagent smart dispensing machines with free HIVST kits on the campus of the school (54). Global evidence indicates that HIVST devices are both convenient and efficient (55–57) and that self-testing reagents have high sensitivity and specificity (58, 59). This underscores HIVST’s potential as a scalable tool for expanding testing coverage globally, particularly in resource-limited or stigma-prone contexts (59).

Our study found that gender significantly influences willingness to self-test for HIV, with females more inclined to undergo testing than males, which contrasts with some previous findings (37, 60), where females were more inclined to undergo HIVST than males in this study. This may be because the percentage of female students participating in HIV prevention education activities in this school is higher than that of male students. Sexual behavior is the main form of HIV transmission. The rate of HIV infection among young Chinese students has risen by over 30% annually in recent years, primarily through transmission among men who have sex with me (61–63). Therefore, it is particularly urgent to increase publicity and education on HIV prevention for male students to effectively curb the spread of HIV in colleges and universities. In this survey, the higher the number of heterosexual friends and sexual partners, the more inclined to conduct HIVST. This suggests that social networks can be an important way to obtain information and influence health behaviors (64), and people are gradually recognizing the increased risk as well as the importance of health. In addition, the attenuated willingness among arts majors (OR = 0.53) warrants attention, potentially reflecting disciplinary subculture norms that merit targeted intervention (65).

To adopt healthy behaviors and change poor behaviors, the Health Belief Model is key (66). HIV awareness, including knowledge of HIV testing, has a positive impact on willingness to self-test for HIV (67, 68) and can reduce the incidence of HIV risky behaviors (69–71). Health education can increase college students’ awareness of HIV and promote the adoption of healthy behaviors to prevent HIV infection (72–74). Our HBM integration demonstrated self-efficacy (*β* = 0.38) and cues to action (β = 0.42) as principal behavioral drivers, while perceived barriers (β = −0.31) constituted the strongest inhibitor - findings corroborated by random forest variable importance rankings, which is consistent with the relevant studies (70, 75–78). For example, self-efficacy was the predictor of HIV testing in Brazilian youth (77), while cues to action (e.g., SMS reminders) increased clinic-based testing uptake by 30% in Kenyan university students (78). Moreover, using the random forest model for prediction, the results showed that self-efficacy and cues to action had the greatest influence on HIVST. Self-efficacy, which indicates that people who believe they are capable of performing a behavior are more likely to engage in that behavior (79). Action cues or motivation are one of the key factors in learning and a determinant of the occurrence of health behaviors (80). People with high self-efficacy are more likely to take action (81).

The above findings emphasize the urgent need to understand and address the factors that influence HIVST, especially in this high-risk population. In recent years, it has become increasingly difficult to prevent and control HIV due to the popularization of the Internet (82). Although the government and various organizations have carried out a great deal of HIV prevention and control publicity and education, the stigma, discrimination, and isolation of HIV-infected people are still relatively serious (83). High-risk groups are forced to conceal their infection status (84). In this context, the Random Forest analysis in our study validated the integration of the Health Belief Model (HBM) and provided a basis for prioritizing HIVST interventions among university students. Our findings highlight three key priorities: firstly, reducing perceived barriers emerged as the top priority, with recommendations to address financial barriers through subsidies or insurance, promote anonymous postal testing to ensure privacy, and conduct anti-stigma campaigns to correct misconceptions about HIV testing. Next, increasing self-efficacy was identified as the second priority, with strategies to enhance students’ confidence in self-testing through video demonstrations, peer education, and step-by-step goal-setting to reduce anxiety and build success experiences. Finally, optimizing action prompts was highlighted as the third priority, suggesting the placement of free testing kits in accessible locations such as health centers and dormitories, and the use of customized SMS or email reminders to stimulate testing intentions, especially around key dates like World AIDS Day. These integrated strategies aim to make HIVST more accessible, less intimidating, and ultimately increase uptake among at-risk students.

The novelty of this study is that it applies the theory of health-related behavior change to explore the factors influencing HIVST among college students at risk, and uses the random forest model to rank the importance of the influencing factors. Nevertheless, there are several limitations in this study. Firstly, the study was conducted exclusively among undergraduates at Xiangnan College, which limits the generalizability of the findings to the broader and more diverse Chinese university environment. Future research should consider replicating the study in multi-center or geographically diverse samples to enhance the external validity of the results. Secondly, the cross-sectional design limited our ability to infer causal relationships between mental constructs and test behaviors. We recommend future longitudinal or intervention-based studies to establish directional relationships. Thirdly, in this study, data on sexual behavior and HIV testing intentions were self-reported, potentially introducing social desirability or recall bias. Future research should consider validating these self-reported data using biomarkers (e.g., HIV antibody tests) and administrative records (e.g., medical HIV test logs). Fourth, during the stratified sampling process, we only stratified students by major and did not account for grade level. While major significantly influences student behavior and perceptions, grade level may also correlate with high-risk behaviors. Not considering grade level could lead to an uneven sample distribution across grades, potentially introducing selection bias. For future studies, we recommend incorporating both major and grade level in stratified sampling to enhance sample representativeness. Fifth, this study was not able to effectively recruit a sufficient number of men who have sex with men (MSM) students for the analyses, limiting our understanding of HIVST intentions in this key high-risk population. Future research should focus on this population. Finally, this study measured some HBM constructs (e.g., perceived severity and susceptibility) with binomial items, while others used Likert scales. Future research should employ consistent psychometric scales to improve result reliability.

## Conclusion

In conclusion, our dual-model approach identifies modifiable predictors (self-efficacy, cues to action) and high-risk subgroups (arts majors, socially active males) for targeted intervention. The integration of accessible testing infrastructure with HBM-informed education programs, particularly addressing male-specific prevention gaps and disciplinary subcultures, could optimize campus HIV control. Subsequent research should explore hybrid machine learning architectures to capture complex behavioral interactions, ultimately advancing precision public health strategies.

## Data Availability

The raw data supporting the conclusions of this article will be made available by the authors, without undue reservation.

## Footnotes

^1^ www.wjx.cn
