# Supplementary material for: Predicting HIV self-testing intentions among Chinese college students: a dual-model analysis integrating health belief constructs and machine learning prioritization
Source: Front Public Health. 2025 Jul 31;13:1596876. doi: 10.3389/fpubh.2025.1596876 (PMC12351388; doi:10.3389/fpubh.2025.1596876)
Supplement: Supplementary file 1 [file Supplementary_file_1.docx]

**Predicting HIV Self-Testing Intentions** **Among** **Chinese College Students: A Dual-Model Analysis Integrating Health Belief Constructs and Machine Learning Prioritization**

**Method Supplementary 1. Details of the Health Belief Model (HBM)**

**Supplementary Tables**

**Table S1.** Specific questions and assignments for each of the HBM dimensions

**Table S2.** Covariance diagnostic results

**Table S3.** Characteristics of participants reporting unprotected sex and willingness to self-test within the next 6 months (n, %)

**Supplementary Figures**

**Figure S1.** Flow chart of the population included in this study

**Figure S2.** The correlations between willingness to HIV self-testing and HBM

**Figure S3.** The error rates and ROC curves for random forest models

**Method Supplementary 1.** **Details of the** **Health Belief Model (HBM)**

The HBM was first proposed by American social psychologists Godfrey Hochbaum and Irwin Rosenstock in the 1950s [1]. It is a critical theory that explains how individual health behavior is affected by psychological factors and mainly emphasizes that the dominant element of individual health behavior is a personal subjective belief [1,2]. The HBM has been adopted as a conceptual framework and extensively evaluated empirically. It can explain health-promoting behavior and the association between health behaviors and psychological activities [3,4]. The basic HBM consists of six elements (susceptibility, seriousness, benefits, barriers, self-efficacy, and cues to action).

(1) Perceived susceptibility

Perceived susceptibility refers to a person’s subjective perception of the risk of acquiring an illness or disease and is a powerful predictor of health behavior prevention in various populations [5-8].

(2) Perceived severity

Perceived severity refers to a person’s subjective judgment of the severity of a poor health outcome, for example, a disease. Some studies have shown that perceived severity is associated with health-related preventive behaviors, such as condom use and COVID-19 and STI prevention behaviors [7-9]. The more serious a disease is, the more preventive it may be.

(3) Perceived benefits

Perceived benefits refer to a person’s perception of the effectiveness of various actions available to reduce the threat of illness or disease (or to cure illness or disease). These articles showed that perceived benefits promote changes in healthy behavior [10-12].

(4) Perceived barriers

Perceived barriers refer to a person’s feelings about obstacles to performing a recommended health action [13-14] and as obstacles to behavior change, which negatively affect health-related behaviors.

(5) Self-efficacy

Self-efficacy refers to the level of a person’s confidence in his or her ability to perform a behavior successfully. The higher the self-efficacy is, the better a disease-preventive behavior is [15-18]

(6) Cues to action

Cues to action are usually the critical “first step” in health action and can be internal (e.g., chest pains and wheezing) or external (e.g., advice from others, illness of family members, and newspaper articles). Studies have shown that cues to action are factors for predicting health-related intentions and behaviors [7,11,19].

Reference:

[1] Tajeri Moghadam M, Raheli H, Zarifian S, Yazdanpanah M. The power of the health belief model (HBM) to predict water demand management: A case study of farmers' water conservation in Iran. J Environ Manage. 2020;263:110388. doi:10.1016/j.jenvman.2020.110388

[2] Finch CF, White P, Twomey D, Ullah S. Implementing an exercise-training programme to prevent lower-limb injuries: considerations for the development of a randomised controlled trial intervention delivery plan. *Br J Sports Med*. 2011;45(10):791-796. doi:10.1136/bjsm.2010.081406

[3] Wong MCS, Wong ELY, Huang J, et al. Acceptance of the COVID-19 vaccine based on the health belief model: A population-based survey in Hong Kong. *Vaccine*. 2021;39(7):1148-1156. doi:10.1016/j.vaccine.2020.12.083

[4] Wong LP, Alias H, Wong PF, Lee HY, AbuBakar S. The use of the health belief model to assess predictors of intent to receive the COVID-19 vaccine and willingness to pay. Hum Vaccin Immunother. 2020;16(9):2204-2214. doi:10.1080/21645515.2020.1790279

[5] Huang Q, Luo L, Xia BQ, et al. Refinement and Evaluation of a Chinese and Western Medication Adherence Scale for Patients with Chronic Kidney Disease: Item Response Theory Analyses. Patient Prefer Adherence. 2020;14:2243-2252. Published 2020 Nov 18. doi:10.2147/PPA.S269255

[6] Khumsaen N, Stephenson R. Beliefs and Perception About HIV/AIDS, Self-Efficacy, and HIV Sexual Risk Behaviors Among Young Thai Men Who Have Sex With Men. AIDS Educ Prev. 2017;29(2):175-190. doi:10.1521/aeap.2017.29.2.175

[7] Huang Y, Yu B, Jia P, et al. Association between Psychological Factors and Condom Use with Regular and Nonregular Male Sexual Partners among Chinese MSM: A Quantitative Study Based on the Health Belief Model. Biomed Res Int. 2020;2020:5807162. Published 2020 Sep 28. doi:10.1155/2020/5807162

[8] Wang Z, Wu X, Lau J, et al. Prevalence of and factors associated with unprotected anal intercourse with regular and nonregular male sexual partners among newly diagnosed HIV-positive men who have sex with men in China. HIV Med. 2017;18(9):635-646. doi:10.1111/hiv.12500

[9] Zimmermann HM, van Bilsen WP, Boyd A, et al. Prevention challenges with current perceptions of HIV burden among HIV-negative and never-tested men who have sex with men in the Netherlands: a mixed-methods study. J Int AIDS Soc. 2021;24(8):e25715. doi:10.1002/jia2.25715

[10] Hu Y, Zhong XN, Peng B, et al. Associations between perceived barriers and benefits of using HIV pre-exposure prophylaxis and medication adherence among men who have sex with men in Western China. BMC Infect Dis. 2018;18(1):575. Published 2018 Nov 15. doi:10.1186/s12879-018-3497-7

[11] Huang ST, Huang JH, Chu JH. Health Beliefs Linked to HIV Pre-Exposure Prophylaxis Use Intention Among Young Men Who Have Sex with Men in Taiwan. AIDS Patient Care STDS. 2021;35(12):474-480. doi:10.1089/apc.2021.0146

[12] Wang Z, Fang Y, Chan PS, et al. Effectiveness of a Community-Based Organization-Private Clinic Service Model in Promoting Human Papillomavirus Vaccination among Chinese Men Who Have Sex with Men. Vaccines (Basel). 2021;9(11):1218. Published 2021 Oct 20. doi:10.3390/vaccines9111218

[13] Patrick R, Jain J, Harvey-Vera A, et al. Perceived barriers to pre-exposure prophylaxis use among HIV-negative men who have sex with men in Tijuana, Mexico: A latent class analysis. PLoS One. 2019;14(8):e0221558. Published 2019 Aug 22. doi:10.1371/journal.pone.0221558

[14] Viera A, van den Berg JJ, Sosnowy CD, et al. Barriers and Facilitators to HIV Pre-Exposure Prophylaxis Uptake Among Men Who have Sex with Men Who Use Stimulants: A Qualitative Study. AIDS Behav. 2022;26(9):3016-3028. doi:10.1007/s10461-022-03633-5

[15] Wang C, Tucker JD, Liu C, Zheng H, Tang W, Ling L. Condom use social norms and self-efficacy with different kinds of male partners among Chinese men who have sex with men: results from an online survey. BMC Public Health. 2018;18(1):1175. Published 2018 Oct 16. doi:10.1186/s12889-018-6090-5

[16] Safren SA, Blashill AJ, Lee JS, et al. Condom-use self-efficacy as a mediator between syndemics and condomless sex in men who have sex with men (MSM). Health Psychol. 2018;37(9):820-827. doi:10.1037/hea0000617

[17] Qu D, Zhong X, Lai M, Dai J, Liang H, Huang A. Influencing Factors of Pre-Exposure Prophylaxis Self-Efficacy Among Men Who Have Sex With Men. Am J Mens Health. 2019;13(2):1557988319847088. doi:10.1177/1557988319847088

[18] Bayır B, Aylaz R. The effect of mindfulness-based education given to individuals with substance-use disorder according to self-efficacy theory on self-efficacy perception. Appl Nurs Res. 2021;57:151354. doi:10.1016/j.apnr.2020.151354

[19] McGarrity LA, Huebner DM, Nemeroff CJ, Proeschold-Bell RJ. Longitudinal Predictors of Behavioral Intentions and HIV Service Use Among Men Who Have Sex with Men. Prev Sci. 2018;19(4):507-515. doi:10.1007/s11121-017-0824-y

| **Table S1. Specific questions and assignments for each of the HBM dimensions** | | |
| --- | --- | --- |
| **Items** | **Question** | **Value Assignment** |
| Perceived susceptibility | Do you think HIV is a preventable disease? | 1=No, 2=Yes |
|  | Do you think that people who seem to be healthy may be living with HIV? | 1=No, 3=Yes |
|  | Do you think AIDS is a threat to you and your family? | 1=No, 4=Yes |
| Perceived severity | Do you think a pregnant woman with HIV can pass the virus on to her child? | 1=No, 5=Yes |
|  | Do you think a healthy person can be infected with HIV through contact with the blood of an HIV-infected person? | 1=No, 6=Yes |
|  | Do you think a healthy person can get HIV from having sex with an AIDS patient? | 1=No, 7=Yes |
|  | Do you think a healthy person can get HIV from sharing syringes with an HIV patient? | 1=No, 8=Yes |
|  | Do you think men who have sex with men are the most affected by AIDS in China? | 1=No, 9=Yes |
|  | Do you think that contracting other sexually transmitted diseases increases the risk of contracting HIV? | 1=No, 10=Yes |
|  | Do you think the use of new drugs (e.g., methamphetamine, ecstasy, ketamine, etc.) increases the risk of HIV infection? | 1=No, 11=Yes |
| Perceived benefits | Getting tested for HIV can lead to earlier detection of HIV infection and better treatment outcomes. | 1-strongly disagree, 2-disagree, 3=fairly, 4=agree, 5=strongly agree |
|  | Getting tested for HIV can protect my sexual partners. | 1-strongly disagree, 2-disagree, 3=fairly, 4=agree, 6=strongly agree |
|  | Getting tested for HIV can make me feel more at ease with myself. | 1-strongly disagree, 2-disagree, 3=fairly, 4=agree, 7=strongly agree |
|  | Getting tested for HIV can enhance trust between my partner and me. | 1-strongly disagree, 2-disagree, 3=fairly, 4=agree, 8=strongly agree |
| Perceived barriers | I don’t know where to go for counseling and testing. | 1-strongly disagree, 2-disagree, 3=fairly, 4=agree, 9=strongly agree |
|  | I am afraid that people will find out I have been tested for HIV. | 1-strongly disagree, 2-disagree, 3=fairly, 4=agree, 10=strongly agree |
|  | I fear testing positive. | 1-strongly disagree, 2-disagree, 3=fairly, 4=agree, 11=strongly agree |
|  | I don’t have time to get tested. | 1-strongly disagree, 2-disagree, 3=fairly, 4=agree, 12=strongly agree |
|  | I don’t think there is a need to get tested for HIV. | 1-strongly disagree, 2-disagree, 3=fairly, 4=agree, 13=strongly agree |
| Self-efficacy | I am sure I will get tested for HIV in the next six months. | 1-strongly disagree, 2-disagree, 3=fairly, 4=agree, 5=strongly agree |
|  | I will take an HIV test even if I feel embarrassed to do so. | 1-strongly disagree, 2-disagree, 3=fairly, 4=agree, 6=strongly agree |
|  | I will take an HIV test even if I am worried about a positive result. | 1-strongly disagree, 2-disagree, 3=fairly, 4=agree, 7=strongly agree |
|  | I will take an HIV test even if others may find out about it. | 1-strongly disagree, 2-disagree, 3=fairly, 4=agree, 8=strongly agree |
| Cues to action | Information about HIV self-testing in school-related literature would motivate me to get tested. | 1-strongly disagree, 2-disagree, 3=fairly, 4=agree, 9=strongly agree |
|  | Information on the internet about the importance of getting tested for HIV would motivate me to get tested. | 1-strongly disagree, 2-disagree, 3=fairly, 4=agree, 10=strongly agree |
|  | The availability of free HIV self-testing facilities at school would motivate me to get tested. | 1-strongly disagree, 2-disagree, 3=fairly, 4=agree, 11=strongly agree |
|  | A family member or friend testing positive for HIV would motivate me to get tested. | 1-strongly disagree, 2-disagree, 3=fairly, 4=agree, 12=strongly agree |

| **Table S2. Covariance diagnostic results** | |
| --- | --- |
| **Variable** | **Variance Inflation Factor** |
| Gender | 1.12 |
| Specialized field | 1.10 |
| Grade | 1.19 |
| Household registration | 1.04 |
| Living expenses | 1.03 |
| Received relevant knowledge on AIDS prevention and treatment | 1.68 |
| Relationship status | 1.13 |
| Sexual orientation | 1.05 |
| Number of sexual partners | 1.08 |
| Perceived susceptibility | 2.35 |
| Perceived severity | 2.50 |
| Perceived benefits | 1.12 |
| Perceived barriers | 1.15 |
| Self-efficacy | 1.56 |
| Cues to action | 1.49 |


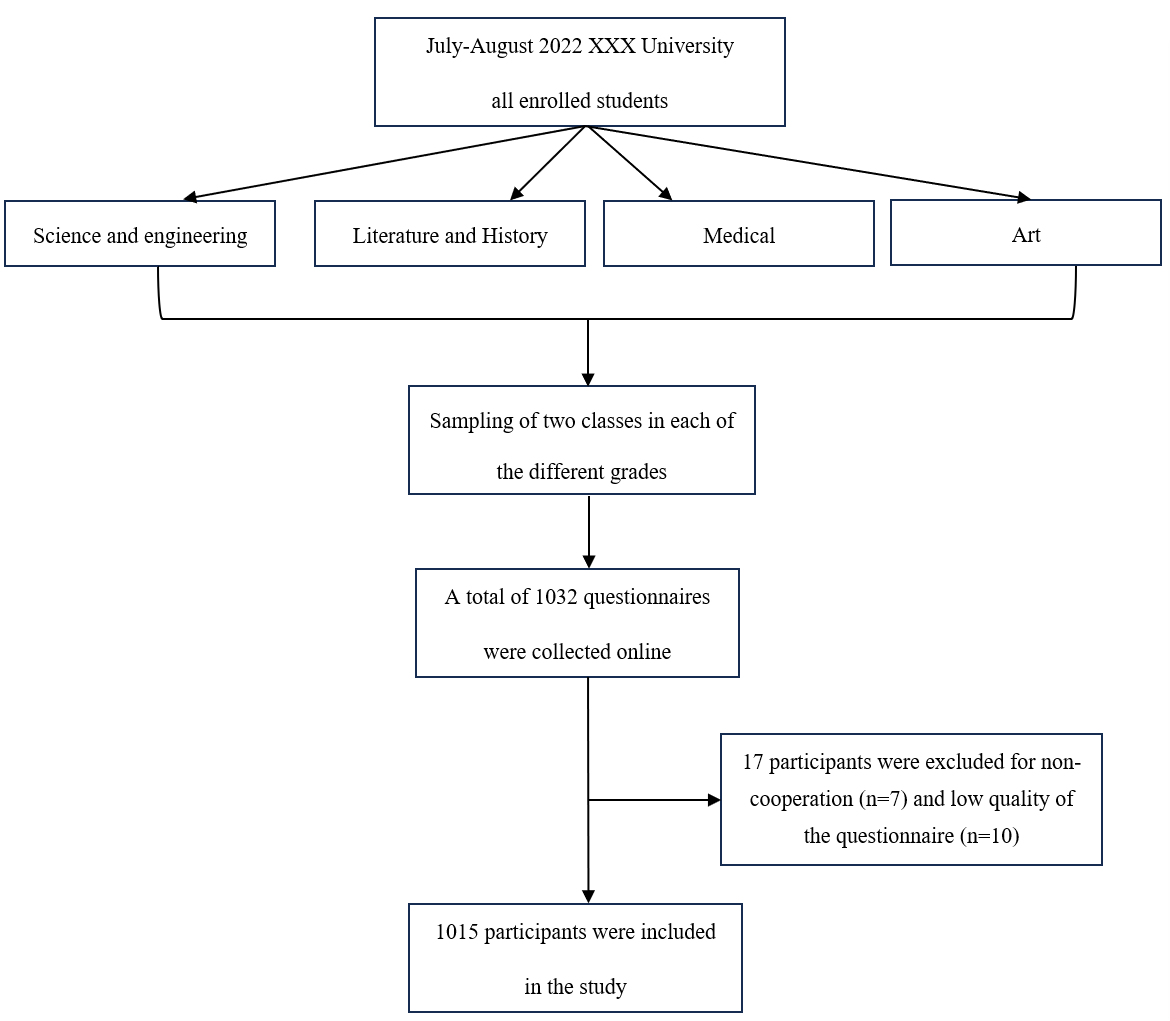
**Figure S1.** **Flow chart of the population included in this study**

**
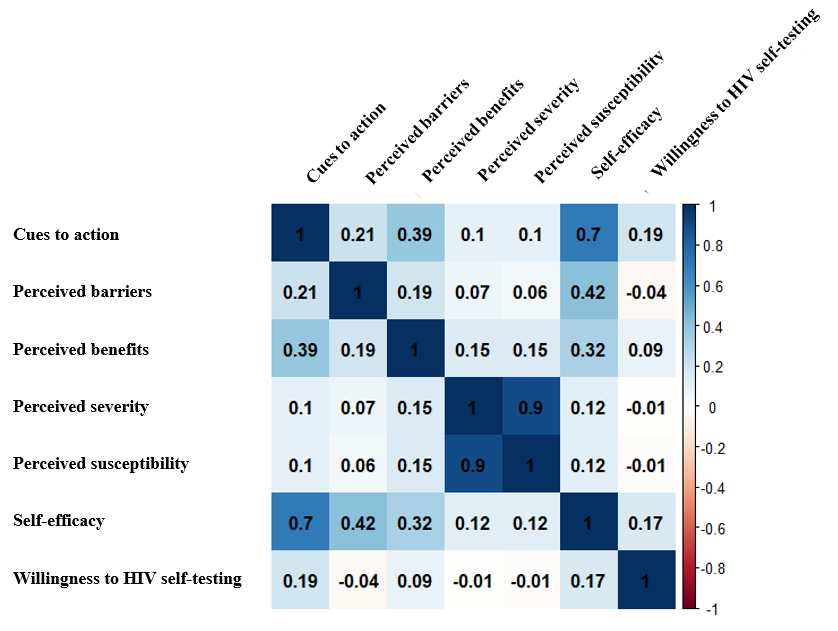
**

**Figure S2. The correlations between willingness to HIV self-testing and HBM**

**(A)**

**
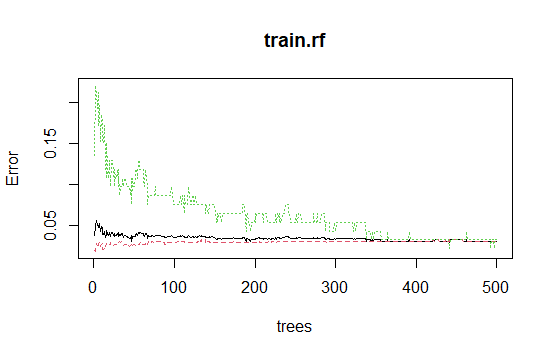
**

**(B)**


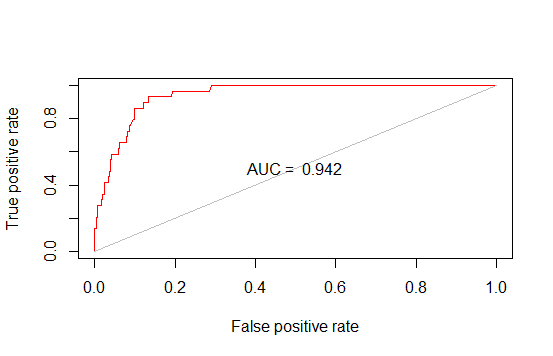


**Figure S3. The error rates and ROC curves for random forest models**

(A) The relationship between the overall error rate and the number of trees

(B) The plot of changes in false positive rate and true positive rate (AUC=0.942)
